# Piglet immunization with a spike subunit vaccine enhances disease by porcine epidemic diarrhea virus

**DOI:** 10.1038/s41541-021-00283-x

**Published:** 2021-02-01

**Authors:** Jieshi Yu, Chithra Sreenivasan, Tirth Uprety, Rongyuan Gao, Chen Huang, Ella J. Lee, Steven Lawson, Julie Nelson, Jane Christopher-Hennings, Radhey S. Kaushik, Eric Nelson, Diego G. Diel, Ben M. Hause, Feng Li, Dan Wang

**Affiliations:** 1 Maxwell H. Gluck Equine Research Center, Department of Veterinary Science, University of Kentucky, Lexington, KY USA; 2 Department of Biology and Microbiology, South Dakota State University, Brookings, SD USA; 3 Department of Veterinary and Biomedical Sciences, Animal Disease Research and Diagnostic Laboratory, South Dakota State University, Brookings, SD USA; 4 Department of Population Medicine and Diagnostic Sciences, Animal Health Diagnostic Center, College of Veterinary Medicine, Cornell University, Ithaca, NY USA

**Keywords:** Microbiology, Diseases

## Abstract

Immunization with an insect cell lysate/baculovirus mixture containing recombinant porcine epidemic diarrhea virus (PEDV) spike protein induced high levels of neutralizing antibodies in both mice and piglets. However, immunization of piglets with this vaccine resulted in enhancement of disease symptoms and virus replication in vaccine recipients exposed to PEDV challenge. Thus, these observations demonstrate a previously unrecognized challenge of PEDV vaccine research, which has important implications for coronavirus vaccine development.

Porcine epidemic diarrhea virus (PEDV), an alphacoronavirus, was first identified in the U.S. in May 2013 from diseased pigs experiencing explosive epidemics of diarrhea and vomiting^1,2^. The virus rapidly spread to most swine-producing states, causing a loss of ~7 million pigs^3^.

Here, we used the standard Bac-to-Bac Baculovirus expression system to produce a recombinant vaccine containing the spike protein of PEDV, designated hereafter Bac-PEDV-S. Similarly, we generated recombinant baculoviruses expressing Ferritin, designated Bac-Ferritin that served as a control. SDS-PAGE with Coomassie-staining and Western-blot assay involving anti-PEDV spike protein antibody identified a predominant protein band in infected *Sf9* cells that migrated as expected for a full-length PEDV spike protein (Fig. 1a–b). Bac-PEDV-S, Bac-Ferritin, inactivated PEDV vaccines and PBS formulated with adjuvant MONTANIDE ISA 50-V2, which were then administered intramuscularly into four groups of 7-week-old BALB/c mice, respectively.

**Fig. 1:**
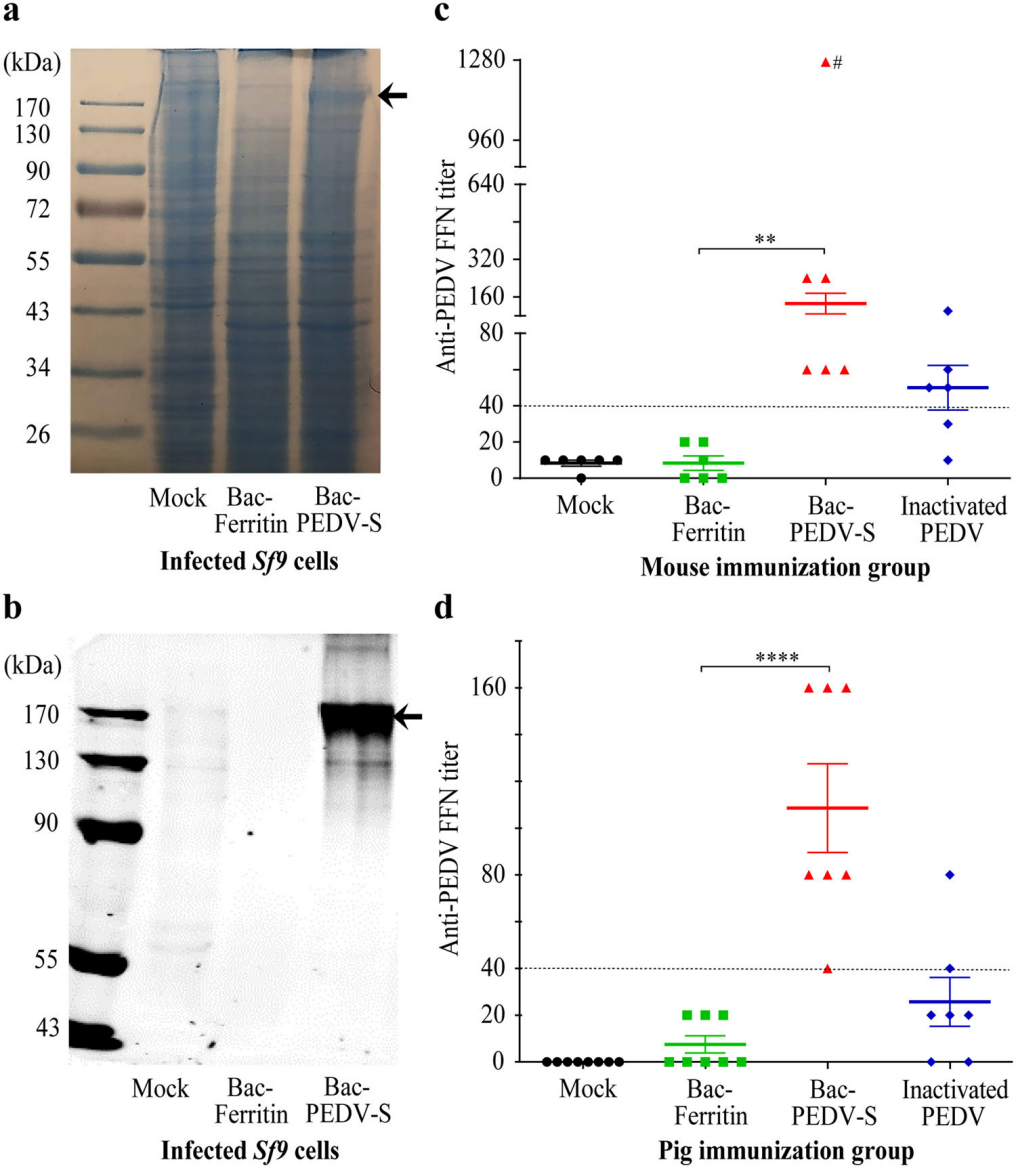
Bac-PEDV-S induced neutralizing antibodies in vaccinated mice and piglets. **a**, **b** The expression of the PEDV S protein (indicated by an arrowhead) was confirmed by SDS-PAGE with coomassie blue staining and Western blotting analysis probed with anti-PEDV S monoclonal antibody. Neutralizing antibodies in mice (**c**) and piglets (**d**) of different treatment groups were evaluated by FFN assay^4^. The meaning of error bars and marked signs were described in the “Methods” section.

Serum samples collected from mice two weeks after the second vaccination were analyzed to determine the immunogenicity of Bac-PEDV-S and designated controls. The standard fluorescent focus neutralization (FFN) assay^4^ was used to measure the titers of virus-neutralizing antibodies in vaccinated animals. Bac-PEDV-S vaccine elicited the mean FFN titer of about 130, which was significantly higher (*p* < 0.01) than that observed in the Bac-Ferritin group (no FFN antibodies detected) (Fig. 1c). The inactivated PEDV vaccine-elicited mean FFN titers of about 50. There were no detectable FFN antibodies in the mock control group (Fig. 1c).

Next, we examined the immunogenicity of the Bac-PEDV-S vaccine in piglets. Analyses of vaccine-induced neutralizing antibody titers in the pre-challenge sera of the vaccinated piglets showed that Bac-PEDV-S vaccinated animals achieved significantly higher levels of virus-neutralizing antibodies when compared to animals in the Bac-Ferritin group (*p* < 0.0001) (Fig. 1d). Specifically, all seven pigs receiving the Bac-PEDV-S vaccine developed moderate neutralizing antibody titers ranging from 40 to 160 with a mean FFN antibody titer of 110. Neither Bac-Ferritin nor mock control elicited any positive virus-neutralizing antibodies in vaccinated piglets. 2/8 animals in the whole-virus inactivated group possessed positive neutralizing antibody titers (40 and 80 respectively) (Fig. 1d).

Despite stimulating high-titer neutralizing antibodies, the Bac-PEDV-S vaccine group exhibited more severe (*p* = 0.09) diarrhea in the majority of vaccinated animals than the Bac-Ferritin group during the observation period after the challenge. Specifically, 3/7 of the vaccinated animals developed moderate to severe diarrhea as early as 2-day post-challenge (dpc) and two animals in this group showed minimal to moderate diarrhea even at 7 dpc (Figs. 2a, c). At 4 dpc, 6/7 vaccinated animals experienced varying levels of diarrhea (diarrhea scores ≥ 1), while only 3/8 animals in the Bac-Ferritin group experienced diarrhea (Fig. 2b). Despite one animal showing diarrhea at 2 dpc in the Bac-Ferritin group (Fig. 2a), no animal showed diarrhea and disease at 7 dpc (Fig. 2c). Moreover, measurements of relative viral loads in fecal samples by RT-qPCR indicated that animals in the Bac-PEDV-S group shed significantly higher levels of the virus than those in the Bac-Ferritin group (*p* < 0.05) (Fig. 2d).

**Fig. 2:**
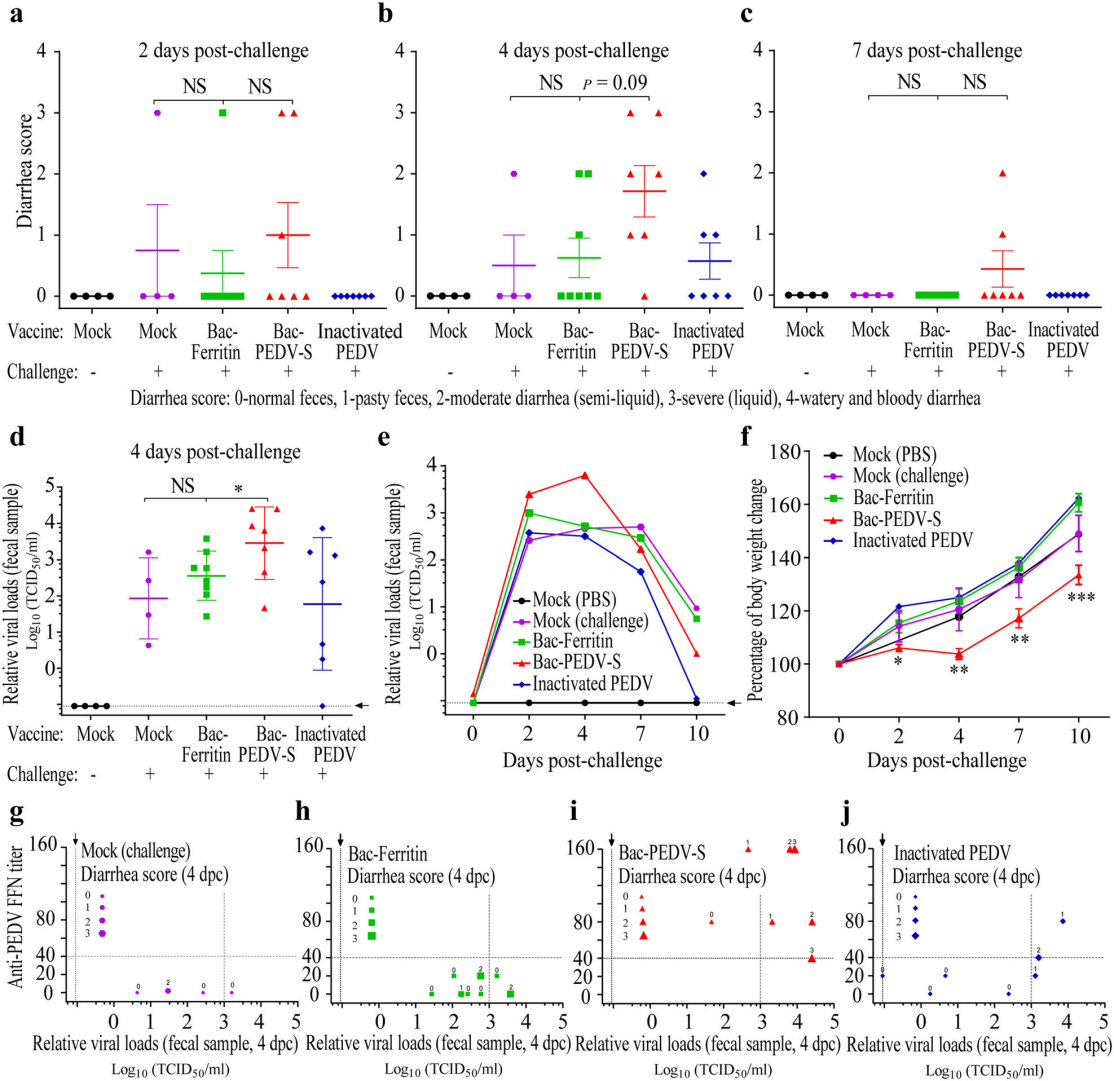
Bac-PEDV-S vaccine group exhibited enhanced clinical symptoms and virus replication. **a–c** Fecal consistency was scored at indicated time points for each group. **d**, **e** PEDV shedding in feces was tested by RT-qPCR and reported as the relative viral loads determined by the standard curve. **f** The averaged body weight gain of piglets on indicated days post-challenge in each group was calculated by comparison with body weight of animals on the day of the challenge. **g**–**j** The neutralizing antibody titers, relative viral loads, and diarrhea scores were plotted together for individual piglets in different vaccination groups. The meaning of error bars and marked signs were described in the “Methods” section.

We also employed a sample pooling/RT-qPCR testing strategy to investigate the fecal virus shedding. The results indicated that the Bac-PEDV-S vaccine enhanced viral shedding especially at 2 and 4 dpc, with elevated levels of fecal virus shedding (i.e., relative viral loads: 2.47 × 10^3^ and 6.29 × 10^3^ TCID_50_/ml, respectively), which was in contrast to relative viral loads (9.86 × 10^2^ and 5.11 × 10^2^ TCID_50_/ml) for the Bac-Ferritin group at these two time-points, respectively (Fig. 2e). Notably, there was a significantly lower (*p* < 0.001) averaged body weight gain (i.e., ~125% of the baseline weight prior to virus challenge) in Bac-PEDV-S vaccine group compared to the Bac-Ferritin group (i.e., ~160% of the baseline weight) during the 11-day observation period after virus challenge (Fig. 2f). Note the unvaccinated and virus challenge group involving four piglets showed characteristics including diarrhea scores, relative fecal virus shedding, the gain of body weight similar to those exhibited in Bac-Ferritin group (Fig. 2a–f). Taken together, these data indicated that Bac-PEDV-S vaccination resulted in a marked enhancement of virus replication and exacerbation of clinical diarrhea and disease in immunized pigs following homologous PEDV challenge.

To further analyze the associations between the neutralizing antibody titers, viral shedding, and clinical signs, we plotted them on individual levels (Fig. 2g–j). Specifically, 6/7 animals vaccinated with the Bac-PEDV-S shed high levels of virus (>10^3^ TCID_50_/ml) and/or experienced diarrhea (diarrhea scores ≥ 1), in spite of the presence of neutralizing antibodies (FFN titers ≥ 40). Nevertheless, only 2/4 animals in the unvaccinated and challenge group, 4/8 animals in the Bac-Ferritin vaccine group and 3/7 animals in the inactivated PEDV vaccine group showed relative viral shedding titers higher than 10^3^ TCID_50_/ml and/or experienced diarrhea (diarrhea scores ≥ 1). In addition, 2/3 animals showing diarrhea scores ≥ 1 and relative viral shedding titers ≥ 10^3^ TCID_50_/ml in the inactivated PEDV vaccine group also had FFN titers ≥ 40. Therefore, we concluded that the neutralizing antibody titers against PEDV were not absolutely associated or shown to be a correlate of protection as demonstrated in this study.

The mechanisms leading to the observed vaccine-induced enhancement of PEDV replication and disease are unknown, however, our observation appears to continue a theme of vaccine-related or antibody-dependent enhancement of infection demonstrated by previous studies, which includes the coronaviruses that cause infectious peritonitis in felines or severe acute respiratory syndrome in humans^5–7^. In light of the overall high levels of PEDV-neutralizing antibody responses elicited by the Bac-PEDV-S vaccine (Fig. 1c–d), it is possible that the observed enhancement of the disease can be attributed to the PEDV spike-specific non-neutralizing antibody response. Considering that the immunogen used in this study was a mixture of insect cell lysate and baculovirus that expressed the PEDV spike proteins, our vaccine preparation likely contained both pre-fusion and post-fusion forms of the PEDV spike. Numerous studies on human coronaviruses including the Middle East respiratory syndrome coronavirus (MERS-CoV) and the severe acute respiratory syndrome coronavirus 2 (SARS-CoV-2) have demonstrated that antibodies targeting the pre-fusion form, not post-fusion form, of the spike protein neutralize virus infection^8–10^. And therefore, we speculate that non-neutralizing antibodies generated in piglets against the post-fusion of PEDV spike protein present in our vaccine may play an important role in the enhancement of clinical diarrhea and virus replication observed in our study, which warrants further investigation. Regardless of the mechanism, this study highlights a previously unrecognized challenge in utilizing the baculovirus-expressed recombinant spike protein vaccine to control PEDV infection of pigs, which has important implications for general coronavirus vaccine development in humans and animals.

## Methods

### Cells and viruses

Vero-76 cells (ATCC CRL-1587) were maintained in Dulbecco’s modified Eagle’s medium (DMEM) containing 10% fetal bovine serum (FBS) at 37 °C with 5% CO_2_. The *Spodoptera frugiperda Sf9* insect cells were purchased from Thermo Fisher Scientific and cultured in the serum-free SF900 II medium (Gibco) in suspension. The PEDV Colorado (PEDV-CO) strain (GenBank: KF272920.1) obtained from the South Dakota Animal Disease Research and Diagnostic Laboratory (ADRDL) was propagated in Vero cells.

### Plasmid construction and recombinant baculovirus production

The Ferritin gene and the coding sequence of a synthetic, codon-optimized full-length spike gene of PEDV-CO strain were cloned into the pFHMSP-LIC-C vector (Addgene Catalog# 26100), respectively, which were conducted by using an In-Fusion HD Cloning Kit (Clontech) according to the manufacturer’s instructions. The resultant plasmids were then transformed into DH10Bac *E. coli* cells (Thermo Fisher Scientific) carrying a baculovirus shuttle vector (bacmid) and a helper plasmid to generate recombinant bacmids. The colonies containing recombinant bacmids with target genes were screened and further validated by PCR and DNA sequencing. Purified recombinant bacmid DNAs were transfected into *Sf9* cells by Cellfectin™ II (Gibco) to generate recombinant baculoviruses, Bac-Ferritin, and Bac-PEDV-S. The virus stocks with high titers were prepared and used to produce the recombinant protein vaccine of interest.

### SDS-PAGE and Western-blotting assay

*Sf9* cells were infected with recombinant baculoviruses at a multiplicity of infection (MOI) of 2. The supernatants were collected, and the cells were lysed. The expression of the PEDV S protein was confirmed by SDS-PAGE with coomassie blue staining and Western-blotting using anti-PEDV S monoclonal antibody (ADRDL). All blots or gels derived from the same experiment and were processed in parallel.

### Preparation of the recombinant spike protein and the whole-virus inactivated vaccines

The recombinant baculovirus vaccines, Bac-PEDV-S and Bac-Ferritin were prepared by infecting *Sf9* cells (1–2 × 10^6^ cells/ml) with the recombinant baculoviruses (at least 1 MOI). At 72–96 h post-infection, 200 ml of infected cells were collected, and the supernatants containing viruses were further concentrated around 100 times by ultracentrifugation at 24,000 rpm for 2 h at 4 °C. The cell pellets were then resuspended by using the concentrated supernatants to make the final volume of about 10 ml. The mixtures were mixed 1:1 (volume per volume, v/v) with adjuvant MONTANIDE ISA 50-V2 (SEPPIC, NJ, USA) to form Bac-PEDV-S and Bac-Ferritin vaccines. Inclusion of the Bac-Ferritin encoding an irrelevant protein to PEDV in this study, prepared under an identical condition, would serve as a control to rule out possible non-specific protective effects derived from baculovirus culture implicated in vaccine efficacy. The whole-virus inactivated vaccine was prepared via the treatment of the PEDV-CO virus (5 × 10^5^ TCID_50_/ml) with 0.02% Beta-propiolactone (BPL) for 20 h at 4 °C. Following the virus replication assay showing no detectable live viruses, the inactivated PEDV-CO was mixed with the above adjuvant to formulate the inactivated vaccine.

### FFN assay

Virus neutralization antibody responses in immunized pigs were measured by using the FFN assay established and performed by the South Dakota ADRDL^4^. This method has been extensively used in the field. Heat inactivated serum samples were two-fold serially diluted in MEM supplemented with 1.5 μg/ml TPCK-treated trypsin and then incubated with 100 foci forming units/100 μl of cell culture adapted PEDV-CO stock at 37 °C for 1 h. After 1 h, the virus-serum mixture was added to Vero cell monolayers and incubated at 37 °C for 2 h, followed by washing with MEM supplemented with 1.5 μg/ml TPCK-treated trypsin. The plates were again incubated for an additional 20–24 h and then fixed with 80% acetone, followed by staining with FITC conjugated mAb against PEDV nucleoprotein (NP) to visualize the infected cells. The highest dilution of the sera showing ≥90% reduction in fluorescent foci compared to negative controls will be the endpoint neutralization titer. Serum samples with a FFN titer <40 were scored negative.

### Assessment of the immunogenicity of the Bac-PEDV-S vaccine in mice

Twenty-four, 6-week-old female BALB/c mice (Jackson Laboratories) were randomly divided into four groups. The mice were given one-week acclimatization before vaccinating with 200 µl of adjuvanted Bac-Ferritin, Bac-PEDV-S, and inactivated PEDV vaccines by intramuscular (IM) route, respectively. The mock group was vaccinated IM with 200 µl of PBS. Booster vaccination with the same dose was administered IM to all groups at 14 days post-vaccination (dpv). Peripheral blood samples were collected from the facial vein at 0 and 14 dpv to evaluate the serum antibody titers. At 28 dpv, all the animals were euthanized by CO_2_ inhalation and blood collected. The immune efficacy of the vaccines was evaluated by estimating the neutralization antibody titers in the sera by FFN assay as described previously^4^. The mice study was approved by the Institutional Animal Care and Use Committee (IACUC) of SDSU (IACUC approval no. 18-052A). The authors have complied with all relevant ethical regulations for animal testing and research.

### Evaluation of the protective efficacy of Bac-PEDV-S vaccine piglets against PEDV challenge

We examined the immunogenicity and protective efficacy of the recombinant Bac-PEDV-S vaccine in pigs. Control groups in this study included Bac-Ferritin, inactivated PEDV, and mock immunization (PBS) groups (with and without virus challenge). This study was approved by the IACUC of SDSU (IACUC approval no. 18-057A). The participants have complied with all relevant ethical regulations for animal testing and research. Neonatal piglets farrowed from PEDV seronegative sows were acquired from Midwest Research Swine. Blood samples were collected from the piglets to confirm the seronegative status before vaccination. The immunogenicity and protective efficacy of 2 ml of Bac-PEDV-S (*n* = 7) and inactivated PEDV (*n* = 8) vaccines, were compared to the Bac-Ferritin (*n* = 8) and mock (*n* = 8) groups. All the piglets were vaccinated with two doses at two-weeks intervals intramuscularly. At 5 weeks of age, the piglets in all vaccination groups as well as in one of the mock immunization (PBS) groups were challenged orally with 2 ml of 2 × 10^5^ TCID_50_/ml of PEDV-CO viruses. The animals were monitored post-infection for clinical symptoms such as diarrhea, vomiting, anorexia, body weight loss, and lethargy. Fecal consistency scoring, body weight measurement, and rectal swab collection were conducted on days 0, 2, 4, 7, and 10 post-challenge (dpc). All the animals were euthanized on 10 dpc. The protective efficacy of the vaccine was evaluated by estimating the neutralization antibody titers in the sera by FFN^4^.

### RT-qPCR analysis of virus RNA in fecal samples

Fecal samples, collected from piglets on 0, 2, 4, 7, and 10 dpc, were pooled together from each group at the indicated time points. The samples collected from piglets on 4 dpc were also tested individually. To make a standard curve, the PEDV-CO virus (3.548 × 10^5^ TCID_50_/ml) was prepared by 10-fold serial dilutions from 10^1^ to 10^6^. Viral RNAs were extracted from these samples and diluted virus standard samples using the MagMAX Viral RNA/DNA Isolation Kit (Life Technologies, Carlsbad, CA). ADRDL performed RT-qPCR assays for these samples according to the manufacturer’s instructions (EZ-PED/TGE/PDCoV MPX 1.0, Tetracore, Rockville, MD). The relative viral loads in feces were determined by plotting their Ct values in the contact of the standard curve. The cut-off Ct value of the RT-qPCR was 38.

### Statistical analysis

The number of animals per group used here is based on the power analysis of the previous results with our vaccine study, which reached statistical power >0.8 at a *p*-value of 0.05. Significant differences in the means of anti-PEDV FFN titers between the Bac-Ferritin and Bac-PEDV-S groups were determined by the student *t*-tests in GraphPad Prism 8.0. The significant differences in diarrhea scores, relative viral loads, and percentage of body weight change between different groups were determined by the one-way ANOVA and Dunnett’s multiple comparisons test in GraphPad Prism 8.0. Values of *p* < 0.05 were considered significant (*p < 0.05; **p < 0.01; ***p < 0.001; ****p < 0.0001). Values of *p* < 0.1 were also marked in figures. In Fig. 1, the dotted line indicated the cutoff FFN antibody value (serum samples with FFN titer ≥ 40 counted as positive). An outlier (#) marked in Fig. 1c was not included in the statistics test. In Fig. 2, dotted lines with a black arrowhead indicated the non-detectable level of PEDV in fecal samples; dotted lines at *y*-axis value across 40 indicated that a FFN titer ≥ 40 was counted as positive; dotted lines at *x*-axis value across 3 indicated high levels of relative viral loads (10^3^ TCID_50_/ml) in fecal samples. Data (Figs. 1c–d, 2a–c, f) on the *y*-axis represent the mean values ± SEM and error bars indicate SEM. Data (Fig. 2d) on the *y*-axis represent the geometric mean with geometric standard deviation (SD) and error bars indicate geometric SD.

### Reporting summary

Further information on research design is available in the Nature Research Reporting Summary linked to this article.

## Supplementary information

Reporting Summary

## Data Availability

The raw data used for the preparation of figures of this study are available from the corresponding author upon reasonable request.

## Supplementary information

**Supplementary information** The online version contains supplementary material available at 10.1038/s41541-021-00283-x.
