# Supplementary material for: Piglet immunization with a spike subunit vaccine enhances disease by porcine epidemic diarrhea virus
Source: NPJ Vaccines. 2021 Feb 1;6:22. doi: 10.1038/s41541-021-00283-x (PMC7851141; doi:10.1038/s41541-021-00283-x)
Supplement: Supplementary file 1 — Reporting Summary [file 41541_2021_283_MOESM1_ESM.pdf]

nature research

Corresponding author(s): NPJVACCINES-00995R

Last updated by author(s): Nov 19, 2020

Reporting Summary

Nature Research wishes to improve the reproducibility of the work that we publish. This form provides structure for consistency and transparency in reporting. For further information on Nature Research policies, see our Editorial Policies and the Editorial Policy Checklist.

Statistics

For all statistical analyses, confirm that the following items are present in the figure legend, table legend, main text, or Methods section.

n/a	Confirmed	
☐	☒	The exact sample size (n) for each experimental group/condition, given as a discrete number and unit of measurement
☐	☒	A statement on whether measurements were taken from distinct samples or whether the same sample was measured repeatedly
☐	☒	The statistical test(s) used AND whether they are one- or two-sided<br>*Only common tests should be described solely by name; describe more complex techniques in the Methods section.*
☒	☐	A description of all covariates tested
☐	☒	A description of any assumptions or corrections, such as tests of normality and adjustment for multiple comparisons
☐	☒	A full description of the statistical parameters including central tendency (e.g. means) or other basic estimates (e.g. regression coefficient) AND variation (e.g. standard deviation) or associated estimates of uncertainty (e.g. confidence intervals)
☐	☒	For null hypothesis testing, the test statistic (e.g. F, t, r) with confidence intervals, effect sizes, degrees of freedom and P value noted<br>*Give P values as exact values whenever suitable.*
☒	☐	For Bayesian analysis, information on the choice of priors and Markov chain Monte Carlo settings
☒	☐	For hierarchical and complex designs, identification of the appropriate level for tests and full reporting of outcomes
☐	☒	Estimates of effect sizes (e.g. Cohen's d, Pearson's r), indicating how they were calculated

Our web collection on statistics for biologists contains articles on many of the points above.

Software and code

Policy information about availability of computer code

Data collection	no software was used
Data analysis	GraphPad Prism 8.0

For manuscripts utilizing custom algorithms or software that are central to the research but not yet described in published literature, software must be made available to editors and reviewers. We strongly encourage code deposition in a community repository (e.g. GitHub). See the Nature Research guidelines for submitting code & software for further information.

Data

Policy information about availability of data

All manuscripts must include a data availability statement. This statement should provide the following information, where applicable:

- Accession codes, unique identifiers, or web links for publicly available datasets
- A list of figures that have associated raw data
- A description of any restrictions on data availability

The raw data used for the preparation of figures of this study are available from the corresponding author upon reasonable request

Field-specific reporting

Please select the one below that is the best fit for your research. If you are not sure, read the appropriate sections before making your selection.

☒ Life sciences ☐ Behavioural & social sciences ☐ Ecological, evolutionary & environmental sciences

For a reference copy of the document with all sections, see nature.com/documents/nr-reporting-summary-flat.pdf

Life sciences study design

All studies must disclose on these points even when the disclosure is negative.

Sample size	The number of animals per group used here was determined using a minimum statistical power of 0.8 and a significant p-value of 0.05.
Data exclusions	An outlier (#) marked in the Figure 1c was not included in the statistics test. This value did not pass the normality test.
Replication	All the data except the body weights and clinical scores were obtained from at least two or three independent experiments. RT-qPCR experiments to measure viral loads with the Ct value as the readout in the context of the standard curve were performed by South Dakota's Animal Disease Research and Diagnostic Laboratory according to the standard protocol, which is certified by AAVLD (American Association of Veterinary Laboratory Diagnosticians).
Randomization	Animals were randomly allocated into different experimental groups.
Blinding	The researchers were blinded to group allocation during data collection and analysis.

Reporting for specific materials, systems and methods

We require information from authors about some types of materials, experimental systems and methods used in many studies. Here, indicate whether each material, system or method listed is relevant to your study. If you are not sure if a list item applies to your research, read the appropriate section before selecting a response.

Materials & experimental systems

n/a	Involved in the study	
☐	☒	Antibodies
☐	☒	Eukaryotic cell lines
☒	☐	Palaeontology and archaeology
☐	☒	Animals and other organisms
☒	☐	Human research participants
☒	☐	Clinical data
☒	☐	Dual use research of concern

Methods

n/a	Involved in the study	
☒	☐	ChIP-seq
☒	☐	Flow cytometry
☒	☐	MRI-based neuroimaging

Antibodies

Antibodies used	Anti-PEDV S monoclonal antibody, provided by South Dakota Animal Disease Research and Diagnostic Laboratory (ADRDL)
Validation	The mouse anti-PEDV S monoclonal antibody was verified by SD-ADRDL.

Eukaryotic cell lines

Policy information about cell lines

Cell line source(s)	Vero-76 cell (ATCC CRL-1587), Spodoptera frugiperda Sf9 insect cell
Authentication	The cell lines used were authenticated by the vendors (ATCC; Invitrogen)
Mycoplasma contamination	The cell lines was tested negative for mycoplasma contamination.
Commonly misidentified lines (See ICLAC register)	N/A

Animals and other organisms

Policy information about studies involving animals; ARRIVE guidelines recommended for reporting animal research

Laboratory animals	6-week-old female BALB/c mice (Jackson Laboratories); Neonatal piglets (Midwest swine research farm)
Wild animals	The study did not involve wild animals
Field-collected samples	The study did not involve samples collected from the field
Ethics oversight	The mice study was approved by the Institutional Animal Care and Use Committee (IACUC) of SDSU (IACUC approval no. 18-052A). The pigs study was approved by the IACUC of SDSU (IACUC approval no. 18-057A).

Note that full information on the approval of the study protocol must also be provided in the manuscript.
